# Time in range, especially overnight time in range, is associated with sudomotor dysfunction in patients with type 1 diabetes

**DOI:** 10.1186/s13098-021-00739-z

**Published:** 2021-10-26

**Authors:** Zhou-qin Feng, Qing-yu Guo, Wei Wang, Yan-yu Yuan, Xu-guang Jin, Hui Zhou, Jun Liu, Hai-yan Lei, Xin-yi Yang, Jun Liu, Bin Lu, Jia-qing Shao, Ping Gu

**Affiliations:** 1 Department of Endocrinology, Jinling Hospital, the First School of Clinical Medicine, Southern Medical University, Nanjing, China; 2 Department of Endocrinology, Jinling Hospital, School of Medicine, Nanjing University, Nanjing, China; 3 Department of Endocrinology, Jinling Hospital, Nanjing Medical University, Nanjing, China

**Keywords:** Time in range, Sudomotor dysfunction, SUDOSCAN, Diabetic peripheral neuropathy, Continuous glucose monitoring, Type 1 diabetes

## Abstract

**Background:**

Time in range (TIR) is advocated as key metric of glycemic control and is reported to be associated with microvascular complications of diabetes. Sudomotor dysfunction is among the earliest detectable diabetic peripheral neuropathy (DPN). We set about to research the relationship between TIR including overnight TIR and sudomotor function detected by SUDOSCAN with the intention of exploring the correlation of TIR including overnight TIR and early DPN in type 1 diabetes (T1D).

**Methods:**

95 patients with T1D were enrolled. TIR including nocturnal TIR of 3.9–10.0 mmol/L was evaluated with CGM. SUDOSCAN measured feet electrochemical skin conductance (FESC) and sudomotor dysfunction was defined as average FESC < 60µS. Logistic regressions were applied to examine the independent association of TIR and overnight TIR with sudomotor function.

**Results:**

The overall prevalence of sudomotor dysfunction was 28.42%. Patients with sudomotor dysfunction had significantly lower TIR for the whole recorded phase and for nighttime. The sudomotor dysfunction prevalence progressively declined with the ascending tertiles of TIR and nocturnal TIR (*P* for trend < 0.05). Correlation analysis showed that the relationship between nocturnal TIR and FESC was stronger than that between TIR and FESC with correlation coefficients were respectively 0.362 and 0.356 (P < 0.001). Finally, logistic regression analysis indicated the independently negative relation between TIR and nocturnal TIR and sudomotor dysfunction (P < 0.05), and the correlation between nocturnal TIR and sudomotor dysfunction was more statistically significant.

**Conclusions:**

TIR is negatively correlated with sudomotor dysfunction in T1D independent of HbA1c. Furthermore, decreased nocturnal TIR is more closely related to the impaired function of sudomotor nerves in sweat glands.

## Background

We are facing an epidemic of diabetes, the number of diabetes worldwide is expected to rise to 700 million by 2045 [1]. As one of the most common microvascular complications of diabetes, diabetic peripheral neuropathy (DPN) is the most critical initial risk factor for diabetic foot and amputation and affects more than 50% of individuals with type 1 diabetes(T1D) or type 2 diabetes(T2D) [2]. According to the World Health Organization, the incidence of lower limb amputation in patients with diabetes is ten times that of non-diabetic patients [3]. It's worth noting that the secretory function of sweat gland, controlled by thin unmyelinated sympathetic C nerve fibers, is pretty vulnerable in the process of diabetes [4]. And sudomotor function, which noninvasively measures early neurophysiologic abnormalities of peripheral nerve fiber via analyzing sweat production or sweat chloride concentrations, have been recommended to mirror early neuropathy by the American Association of Clinical Endocrinologists guidelines in 2015 [5]. Thus, early identification and intervention are the best way to prevent or halt DPN and its devastating sequelae.

Hyperglycemia accounts for the occurrence of neuropathy in patients with T1D [6], hence needing good glycemic control for these people. Usually, we take hemoglobin A1c (HbA1c) as the standard for glucose. However, people with T1D are known to have fluctuating blood sugar, so HbA1c may be unreliable for the reason that it misses the information about hypoglycemia, glycemic variability or the daily mode of blood sugar [7].

Continuous glucose monitoring (CGM) technologies provide the preferable mean to capture glucose trends and glucose variability than HbA1c nowadays. Among CGM derived metrics, ‘time in ranges’ encompassing time in target range of 3.9–10 mmol/L (TIR), time above range of 10 mmol/L (TAR) as well as time below range of 3.9 mmol/L (TBR) are in the spotlight. ‘Time in ranges’ can directly reflect short-run glycemic control [8] and published data revealed a substantial correlation between TIR and HbA1c [9, 10]. Based on extensive clinical evidence, American Diabetes Association guidelines for 2020 advocated TIR should be accepted as a clinical endpoint and an emerging metric for assessment of glycemic control [11]. TIR complements information obtained from HbA1c measurement for identifying patients who have the risk of microvascular complications of diabetes [12], whereas there is little report on the impact of TIR on early DPN in T1D. Moreover, to our knowledge, studies on nocturnal TIR and diabetic complications are lacking, and nocturnal glycemic control has been proven to be associated with adverse diabetic outcomes [13, 14].

SUDOSCAN (Impeto Medical, Paris, France) measures sudomotor function quantitatively, and this approach has the advantages of safety, high efficiency and objectivity [15]. The experiment is based on an electrochemical reaction between chlorine ions in sweat and the nickel electrode plates on which the subject’s hands and feet are placed. After stimulating by a low voltage (< 4 V), the attracted chloride ions begin to move towards the plates, generating a current. Then, the device output the electrochemical skin conductance (ESC) by automatically calculating the ratio of derivative current to the applied low-voltage [4]. Here, we set about to research the relationship between TIR including overnight TIR and sudomotor function detected by SUDOSCAN with the intention of exploring the correlation of TIR including overnight TIR and early DPN in T1D.

## Materials and methods

### Research objects

We included 95 individuals with T1D who were hospitalized to better control their blood sugar. These patients both wore 72-h blind CGM (Meiqi Corporation) and underwent sudomotor function test during their hospitalization at the endocrinology department of the Jinling Hospital, the First School of Clinical Medicine, Southern Medical University from October 2018 to July 2020. T1D was diagnosed in line with the criteria of World Health Organization (WHO) in 1999. At the time of study, these patients followed their usual regimen of insulin pump or multiple daily subcutaneous insulin injection (MDSI). Our subjects did not take drugs that could have an effect on the sympathetic system (such as beta or alpha blockers and antineoplastic drugs), had no history of electronic implants or foot ulcer, and did not suffer from severe skin disease involving the soles and palms. Exclusion criteria included: (1) An acute condition requiring intervention such as ketoacidosis, hypoglycemic coma; (2) Secondary neurological impairment result from drugs, alcoholism, vitamin B12 deficiency or thyroid disease and so on. We got approved from local ethics committee and all participants were given informed consent.

### Clinical and biochemical information

We obtained information of general clinical characteristics from the electronic medical records system. Physical measurements consist of age, gender, diabetes duration, height, weight, Systolic blood pressure(SBP), diastolic blood pressure (DBP), diabetic complications, and status of insulin administration. Body mass index (BMI) was calculated as weight (kg)/height (m)^2^. Laboratory examinations including hemoglobin A1C (HbA1c), triglyceride (TG), total cholesterol (TC), high-density lipoprotein (HDL), low-density lipoprotein (LDL), serum albumin (Alb), serum urea nitrogen (BUN), and serum creatinine (Scr), uric acid (UA). HbA1c was determined by high performance liquid chromatography (HLC-723G8 Automatic glycosylated hemoglobin Analyzer, TOSOH, Japan). All the above biochemical indices were tested under fasting conditions for more than 10 h. Previous or present smokers were considered to have smoked. Criteria for diagnosing diabetic kidney disease (DKD) was based on urinary albumin-to-creatinine ratio (ACR) being over 30 mg/g by at least two examinations. Diabetic retinopathy (DR) was determined by the same ophthalmologist through fundus examination and stereofundus photography after pupillary dilation.

### CGM metrics calculation

During the wearing-period of blind CGM for three days, a skillful nurse was informed to correct the instrument via entering capillary blood glucose measurements more than four times per day. 288 continuous glucose values were recorded daily. After 72-h recording, all data were acquired retrospectively. Software Easy GV Version 9.0R2 from Oxford University was used to calculate the measurements representing blood glucose variability (GV), including M value, average daily risk range (ADDR), amplitude of glucose excursions (MAGE), standard deviation (SD), and coefficient of variation (CV). TIR represented the percentage of time blood sugar reached the level of 3.9–10 mmol/L. TBR represented the percentage of time blood sugar below the level of 3.9 mmol/L. TAR represented the percentage of time blood sugar above the level of 10 mmol/L. Nocturnal ‘time in ranges’ were defined as percentage of time blood sugar reached the corresponding level between 00:00 and 6:00 A.M (6 h). Our calculations were the inclusion of all data within 72 h.

### Sudomotor function evaluation

On the afternoon of the hospitalization the sudomotor testing was done. SUDOSCAN (Impeto Medical, Paris, France) consists of two sets of nickel electrodes for the hands and feet that are connected to a computer for recording and data output. At machine start, patients were told to place their palms of their hands and the soles of their feet on the electrodes and a current incremental voltage of 4 V is applied after around 2–3 min. The main parameters output from the machine that we care are ESC of hands and feet. Lower ESC value (use μS said) means worse sudomotor function. As recommended by the manufacturer, skin conductance is considered abnormal if it is below 60µS in the feet. Several clinical studies led to further confirmation of the 60μS thresholds by comparing ESC scores against multiple validated DPN tools [16–18]. Accordingly, we set the feet ESC (FESC) equal to 60 μS as the cut-off point for grouping [19]. So the study patients were divided into normal sudomotor function group (FESC ≥ 60µS) and sudomotor dysfunction group (FESC < 60µS).

### Statistical analysis

Data analysis were performed with SPSS 25.0. Continuous data with normal distribution were expressed in mean ± SD, those with skewed distribution were expressed in median (interquartile). Categorical data were expressed in count (percentages). Between-group comparisons were achieved by Student’s t-test, the Mann–Whitney test, and the chi-squared test. In the light of the distribution characteristics of the data, Pearson correlation analysis and Spearman correlation analysis were separately adopted to identify the relationships between variables. The binary logistic regression analysis and the linear regression analysis with FESC as a categorical variable or a continuous variable were applied to examine the independent correlation between TIR and sudomotor function. Odds ratios (ORs) and 95% confidence intervals were listed. *P* < 0.05 was of statistically significance.

## Results

### Clinical characteristics of the study objects

Our study of the 95 patients with T1D, 74.74% were male, showed a mean age of 36(27,52) years, mean BMI of 21.77 ± 3.11 kg/m^2^, median diabetes course of 4 (1,10) years, mean HbA1c level of 9.78 ± 2.44%. The overall incidence rate of sudomotor dysfunction was prevalent in 28.42% of enrolled patients. As illustrated by Table 1, the majority of baseline characteristics of subjects in the sudomotor dysfunction group paralleled those of the normal sudomotor function group except for blood urea nitrogen, serum albumin and the prevalence of diabetic kidney disease (DKD). Patients with sudomotor dysfunction had a higher level of blood urea nitrogen, a bigger proportion of suffering from DKD and a lower level of serum albumin (all *P* < 0.05).

**Table 1:** Characters of all the patients and of patients grouped by sudomotor function

	Total (N = 95)	sudomotor dysfunction (+) group (N = 27)	sudomotor dysfunction (−) group (N = 68)	*P* value
Age (y)	36.00(27.00,52.00)	35.50(25.25,51.75)	39.00(31.00,54.00)	0.256
Male (n, %)	71(74.74%)	21(77.78%)	50(73.53%)	0.667
BMI (kg/m^2^)	21.77 ± 3.11	21.06 ± 3.27	22.06 ± 3.03	0.160
DM duration (y)	4.00(1.00,10.00)	7(0.5,14.00)	4.00(1.00,8.00)	0.252
SBP (mmHg)	126.00(120.00,132.00)	130.00(120.00,140.00)	125.00(119.25,131.00)	0.222
DBP (mmHg)	76.00(70.00,83.00)	75.00(70.00,87.00)	76.50(71.00,83.00)	0.987
BUN (mmol/L)	5.77 ± 2.28	6.71 ± 3.51	5.40 ± 1.44	**0.012**
Scr (µmol/L)	56.50(45.18,66.85)	59.40(46.00,73.70)	56.00(44.50,65.00)	0.460
UA (µmol/L)	280.63 ± 107.42	265.33 ± 104.78	286.89 ± 108.65	0.382
TC (mmol/L)	4.43 ± 0.97	4.21 ± 0.83	4.52 ± 1.01	0.166
TG (mmol/L)	0.93(0.65,1.33)	0.97(0.69,1.46)	0.88(0.65,1.24)	0.610
HDL(mmol/L)	1.36 ± 0.40	1.30 ± 0.39	1.39 ± 0.41	0.394
LDL(mmol/L)	2.62 ± 0.81	2.50 ± 0.78	2.67 ± 0.82	0.364
Alb (g/L)	38.00(34.30,40.80)	36.30(31.30,39.30)	38.30(35.98,41.53)	**0.014**
HbA1c (%)	9.78 ± 2.44	9.94 ± 2.79	9.70 ± 2.30	0.672
Smoking (%)	30(31.58%)	12(44.44%)	18(26.47%)	0.089
Retinopathy (n, %)	20(21.05%)	8(29.63%)	12(17.65%)	0.196
DKD (n, %) Treatment (n, %) insulin pump MDSI	15(15.79%) 48(50.53%) 47(49.47%)	8(29.63%) 14(53.85%) 13(48.15%)	7(10.29%) 34(53.13%) 34(50.00%)	**0.043** 0.871

Data are presented as means ± SD, median (25% and 75%interquartiles), and count (percentages) according to characteristics of distribution. Between-group comparisons were conducted by Student’s t-test, the Mann–Whitney test, and the chi-squared test

*BMI* body mass index, *SBP* systolic blood pressure, *DBP* diastolic blood pressure, *BUN* blood urea nitrogen, *Scr* serum creatinine, *UA* uric acid, *TC* total cholesterol, *TG* triglyceride, *HDL* high-density lipoprotein, *LDL* low-density lipoprotein, *Alb* serum albumin, *HbA1c* hemoglobin A1c, *DKD* diabetic kidney disease, *MDSI* multiple daily subcutaneous injection

Glycemic parameters were enumerated in the Table 2. Subjects in sudomotor dysfunction group had significantly lower TIR and higher TAR for the whole recorded phase and for nighttime, as well as higher mean glucose, M value and ADDR. Subjects without sudomotor dysfunction had higher TBR and overnight TBR (all *P* < 0.05).

**Table 2:** CGM glucometrics according to sudomotor function

CGM glucometrics	Total (N = 95)	sudomotor dysfunction (+) group (N = 27)	sudomotor dysfunction (−) group (N = 68)	*P* value
Whole recorded phase (3 days)				
TBR (%)	0.00 (0.00,1.35)	0.00(0.00,0.00)	0.11 (0.00, 2.26)	**0.005**
TIR (%)	46.41 ± 24.27	36.25 ± 25.08	50.44 ± 22.90	**0.009**
TAR (%)	49.32 ± 25.85	60.19 ± 26.72	45.00 ± 24.37	**0.009**
Mean (mmol/L)	10.64 ± 2.60	11.93 ± 3.10	10.13 ± 2.20	**0.009**
M value (mmol/L)	16.56 (9.20,33.18)	23.70(13.15,59.56)	14.56(8.84, 27.00)	**0.024**
ADDR	35.17 ± 14.8	41.41 ± 18.55	32.66 ± 12.37	**0.030**
MAGE (mmol/L)	5.09 ± 2.49	4.95 ± 2.49	5.14 ± 2.51	0.735
SD (mmol/L)	3.62 ± 1.28	3.72 ± 1.15	3.58 ± 1.33	0.636
CV (%)	34.58 ± 11.35	32.06 ± 10.70	35.59 ± 11.53	0.174
Overnight phase (0:00–6:00 am)				
TBR (%)	0.00(0.00, 3.33)	0.00(0.00, 0.00)	0.00(0.00, 5.63)	**0.013**
TIR (%)	64.72(34.17, 88.33)	38.61(2.22, 69.72)	67.64(48.26, 90.35)	**0.004**
TAR (%)	33.33(5.28, 57.22)	59.72(18.44, 97.98)	29.72(2.85, 47.64)	**0.001**

Data are presented as means ± SD and median (25% and 75%interquartiles) according to characteristics of distribution. Between-group comparisons were conducted by Student’s t-test and the Mann–Whitney test

TBR, time below range; TIR, time in range; TAR, time above range; Nocturnal TIR was defined as percentage of time blood sugar reached the level of 3.9–10 mmol/L between 00:00 and 6:00 A.M (6 h). Nocturnal TBR was defined as percentage of time blood sugar below the level of 3.9–10 mmol/L between 00:00 and 6:00 A.M (6 h). Nocturnal TAR was defined as percentage of time blood sugar above the level of 3.9–10 mmol/L between 00:00 and 6:00 A.M (6 h)

*ADDR* average daily risk range, *MAGE* amplitude of glucose excursions, *SD* standard deviation, *CV* coefficient of variation

### Prevalence of sudomotor dysfunction among groups according to tertiles

**Fig. 1:**
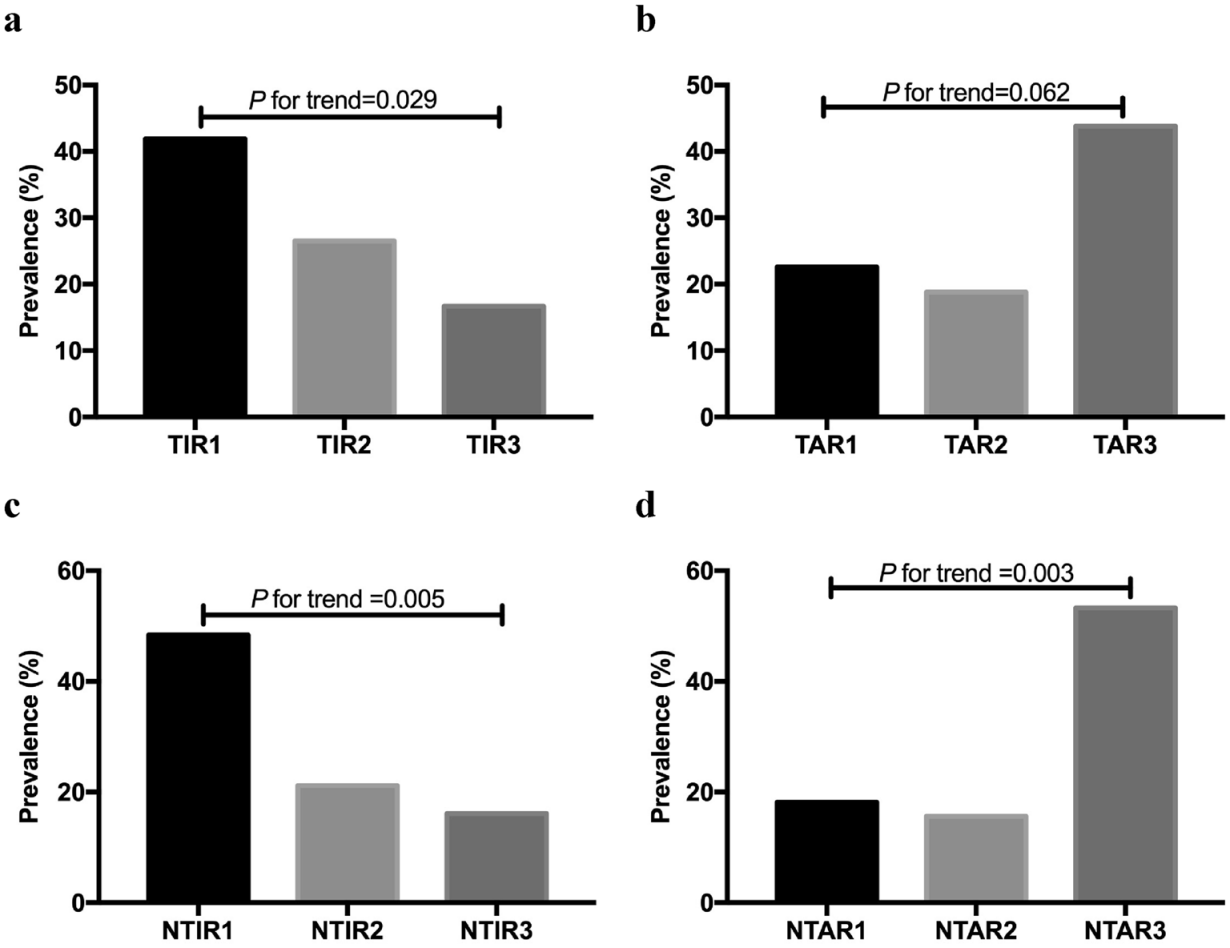
Prevalence of sudomotor dysfunction in tertiles of time in range (**a** TIR1: TIR < 35.53%; TIR2 :35.53% ≤ TIR≤58.86%; TIR3: TIR > 58.86%), time above range (**b** TAR1: TAR < 37.74%; TAR2 :37.74% ≤ TAR ≤ 60.43%; TAR3: TAR > 60.43%), nocturnal time in range (**c** NTIR1: nocturnal TIR < 47.94%; NTIR2: 47.94% ≤ nocturnal TIR≤83.92%; NTIR3: nocturnal TIR > 83.92%) and nocturnal time above range (**d** NTAR1: nocturnal TAR < 12.50%; NTAR2 :12.50% ≤ nocturnal TAR ≤50.00%; NTAR3: nocturnal TAR > 50.00%). Linear-by-linear association chi-square test was applied to determine the P value for trend

Because of the extremely low frequency of TBR and overnight TBR in our research, we just included TIR, TAR, nocturnal TIR and nocturnal TAR for analysis. As shown in Fig. 1, these target metrics were all stratified by tertiles. As for TIR and nocturnal TIR, the prevalence of sudomotor dysfunction decreased with increasing tertiles (all *P* for trend < 0.05). The sudomotor dysfunction prevalence was higher in patients in the first tertile of TIR (41.9%) than that in the second (26.5%) and the third (16.7%). Prevalence rates from the bottom tertile to the top tertile of nocturnal TIR were 48.4%%, 21.2%, and 16.1%. Picture b and d depicted the prevalence of sudomotor dysfunction was higher in the highest tertile of TAR (43.8%) and nocturnal TAR (53.3%) than in the lower tertiles of TAR and nocturnal TAR.

### Correlation between CGM data and the other clinical index and ESC

Through the Pearson correlation analysis and the Spearman’s correlation analysis, we found a significant positive correlation between TIR, TBR, nocturnal TIR and nocturnal TBR and FESC (r = 0.356, r_s_ = 0.356,0.397,0.362,0.311, respectively, *P* < 0.05). TAR and nocturnal TAR were found to negative associated with FESC (r = − 0.391, r_s_ = − 0.405, respectively, *P* < 0.05). HESC showed an inverse correlation with TAR (r = − 0.208, *P* < 0.05). M value and ADDR also had obviously negative concerning with FESC (r_s_ = − 0.307, r = − 0.375, respectively, *P* < 0.05). Serum albumin was positively associated with FESC and HESC (r_s_ = 0.271, 0.248, respectively, *P* < 0.05). DKD was negatively associated with FESC and HESC (r = − 0.281, − 0.246, respectively, *P* < 0.05). There was no influence of blood urea nitrogen had on FESC and HESC (Table 3).

**Table 3:** Correlations of glycemic metrics and other clinical variables and ESC

	FESC		HESC	
	*r*/*r*_*s*_	*P*	*r*/*r*_*s*_	*P*
TBR	0.397	< 0.001	0.185	0.073
TIR	0.356	< 0.001	0.189	0.067
TAR	− 0.391	< 0.001	− 0.208	0.043
Noctural TBR	0.311	0.002	0.104	0.315
Noctural TIR	0.362	< 0.001	0.156	0.132
Noctural TAR	− 0.450	< 0.001	− 0.177	0.086
M value	− 0.307	0.002	− 0.185	0.072
ADDR	− 0.375	< 0.001	− 0.157	0.131
BUN	− 0.168	0.110	− 0.191	0.068
Alb	0.271	0.008	0.248	0.016
DKD	− 0.281	0.006	− 0.246	0.016

Pearson (r) or Spearman (r_s_) correlations were used to investigated associations between variables

TBR, time below range; TIR, time in range; TAR, time above range; Nocturnal ‘time in ranges’ were defined as percentage of time blood sugar reached the corresponding level between 00:00 and 6:00 A.M (6 h); FESC, feet electrochemical skin conductance; HESC, hand electrochemical skin conductance; ADDR, average daily risk range; BUN, blood urea nitrogen; Alb, serum albumin; DKD, diabetic kidney disease

### Binary logistic regression analysis of FESC as a categorical variable

Table 4 interpreted that TIR and overnight TIR were significantly associated with risk of abnormal sudomotor function on binary logistic regression analysis. The ORs for the risk of sudomotor dysfunction were respectively 0.974(95%*CI*:0.955–0.994) and 0.977(95%*CI*:0.963–0.992) per 10% increase in TIR and overnight TIR in the crude model. After adding compounding variables including sex, age, diabetes duration, BMI, Alb, smoking, BUN, and DKD, the relationship between TIR and overnight TIR and sudomotor dysfunction remained (all *P* < 0.05). We added HbA1c in the model 3 of the binary logistic regression, the similar result was observed (*P* < 0.05). More statistically significant results were obtained between nocturnal TIR and sudomotor dysfunction. Apart from that, M value was positively associated with risk of abnormal sudomotor function in both the pre-and post-adjustment models. ADDR was statistically associated with sudomotor dysfunction only in the crude logistic model, as shown in the Table 4.

**Table 4:** Glycemic parameters on risk of sudomotor dysfunction

Parameters	*OR*	*95%CI*	*P*
TIR			
Crude	0.974	0.955 to 0.994	0.012
Model 1	0.975	0.953 to 0.998	0.031
Model 2	0.975	0.951 to 0.999	0.041
Model 3	0.975	0.951 to 1.000	0.047
Nocturnal TIR			
Crude	0.977	0.963 to 0.992	0.002
Model 1	0.979	0.963 to 0.996	0.016
Model 2	0.979	0.961 to 0.997	0.024
Model 3	0.980	0.962 to 0.998	0.028
M value			
Crude	1.036	1.012 to 1.061	0.003
Model 1	1.041	1.012 to 1.071	0.005
Model 2	1.040	1.010 to 1.070	0.008
Model 3	1.042	1.012 to 1.074	0.007
ADDR			
Crude	1.041	1.009 to 1.074	0.012
Model 1	1.036	0.999 to 1.075	0.054
Model 2	1.036	0.998 to 1.075	0.064
Model 3	1.039	0.999 to 1.080	0.056

Crude was not adjusted;

Model 1 was adjusted for sex, age, diabetes duration, BMI, Alb, smoking;

Model 2 was adjusted for all variables in Model 1 plus BUN, DKD;

Model 3 was adjusted for all variables in Model 2 plus HbA1c;

### Multiple liner regression analysis of FESC as a continuous variable

Liner regression analysis described a stable linear association of TIR and nocturnal TIR with FESC. TIR as well as nocturnal TIR were positively correlated with FESC in both the crude and adjusted models including sex, age, diabetes duration, BMI, Alb, smoking, BUN, DKD and HbA1c (all *P* < 0.05) (Table 5). More statistically significant results were obtained between nocturnal TIR and FESC.

**Table 5:** Association of TIR and overnight TIR with FESC by linear regressions

	Models	*β*	*95%CI*	*P*
TIR	Crude	0.359	0.165 to 0.553	< 0.001
	Model 1	0.329	0.132 to 0.527	0.001
	Model 2	0.306	0.1187 to 0.496	0.002
	Model 3	0.263	0.063 to 0.462	0.011
Nocturnal TIR	Crude	0.317	0.175 to 0.458	< 0.001
	Model 1	0.253	0.106 to 0.400	0.001
	Model 2	0.246	0.099 to 0.393	0.001
	Model 3	0.226	0.073 to 0.378	0.004

Crude was not adjusted;

Model 1 was adjusted for sex, age, diabetes duration, BMI, Alb, smoking;

Model 2 was adjusted for all variables in Model 1 plus BUN, DKD;

Model 3 was adjusted for all variables in Model 2 plus HbA1c;

## Discussion

In our study cohort of 95 individuals with T1D, patients with sudomotor dysfunction had features of lower TIR and nocturnal TIR, higher TAR and nocturnal TAR in comparison with patients without sudomotor dysfunction. Group patients by tertiles of TIR and nocturnal TIR, we found the prevalence of sudomtor dysfunction decreased with increasing tertiles. Correlation analysis revealed that the relationship between nocturnal TIR and FESC was stronger than that between TIR and FESC with correlation coefficients were respectively 0.362 and 0.356. Multiple regression analysis revealed that both TIR and nocturnal TIR were inversely associated with the risk of sudomotor dysfunction detected by SUDOSCAN regardless of sex, age, diabetes duration, BMI, Alb, smoking BUN, DKD and HbA1c, and the correlation between nocturnal TIR and sudomotor dysfunction was more statistically significant.

DPN is characterized by broad spectrum of clinical presentations and can stay asymptomatic for a long time, that hinder the prompt diagnosis of this disease [20]. And meanwhile, it is sad to say that the measures we use for detection of DPN in routine clinical practice either are crude, or detect the disease very late when DPN has been well established. For example, scored clinical assessments such as the Michigan Neuropathy Screening Instrument (MNSI), the Neuropathy Impairment Score (NIS), and the Neuropathy Disability Score (NDS), remain subjective, heavily reliant on the examiners’ interpretations [21]. Besides, tests purport to diagnose DPN—including the 10 g monofilament, the Ipswich Touch Test, and vibration perception threshold testing—are still dependent on patients’ subjective response [22]. Although nerve conduction studies (NCS) are the current sensitive and specific tool for DPN, they are not only labour intensive, time consuming, and costly, but are also just an assessment of large nerve fibers function [23].

Small nerve fibers account for 70–90% of the peripheral nerve fibers, and skin biopsies by assessing intraepidermal nerve fiber density (IENFD) shows that small nerve fibers are the first to be damaged, including nerve fibers that innervate sweat glands [24]. Thus, sudomotor function represents an attractive tool to evaluate early neuropathy in people with diabetes. SUDOSCAN is an FDA approved point-of-care device which provides a quantitative measurement of sudomotor function within 3 min [25]. Selvarajah et al. assessed the diagnostic performance of SUDOSCAN for DPN in T1D. Their study defined DPN in terms of established American Academy of Neurology consensus criteria using NCS and clinical examinations and found that the sensitivity and specificity of FESC were respectively 87.5% and 76.2% with the area under the ROC curve (AUC) was 0.85 [26]. Jin et al. also explored the diagnostic efficiency of SUDOSCAN in Chinese with a sensitivity and specificity of 85.6% and 76.2% (AUC = 0.859) [27]. In addition, study also showed the robust repeatability and reproducibility of this machine in person with or without diabetes [28].

A series of large studies evidenced that blood sugar management, as represented by HbA1c, is an effective method for reducing the occurrence or progression of DPN in T1DM. During the mean 6.5-year follow-up, the Diabetes Control and Complications Trial (DCCT) concluded the prevalence of DPN increased substantially in the conventional participants (from 5 to 17%) and only slightly among the intensive group participants (from 7 to 9%) with HbA1c values between the two groups were 9.1% and 7.4%, respectively. Adjusting for the presence of confirmed DSP at baseline, the risk reduction for incident DSP with intensive glucose control during DCCT was 64% [15]. The European Diabetes (EURODIAB) Prospective Complications Study confirmed that HbA1c level during follow-up contributed especially to the risk of neuropathy, independently of the baseline HbA1c [29]. However, we should not ignore the reality that in DCCT the cumulative incidence of neuropathy (15% to 21%) and abnormal nerve conduction (40% to 52%) remained substantial [30]. Besides, through 14 years of the Epidemiology of Diabetes Interventions and Complications (EDIC) study, 25% of subjects in the former intensive treatment group and 35% of subjects in the former conventional therapy group developed confirmed DPN [31]. Such thought-provoking results hint neuropathy can develop even the ‘ideal glycemic control’ tested by HbA1c.

HbA1c estimates blood glucose concentrations over 2–3 months, but it fails to predict the day-to-day glucose excursions [10]. Beck et al. re-analyzed the DCCT’s data to search for the role TIR played in diabetic microvascular outcomes. They found TIR was higher in the intensively treated group than in the conventionally treated group (52 vs. 31%), the per 10% decrease in TIR led to a 64% raise in retinopathy and a 40% increase in microalbuminuria, though resources are a 7-point glucose profile from capillary blood [7]. Our study showed that abnormal sudomotor function group had lower TIR and nocturnal TIR, higher TAR, nocturnal TAR, mean glucose, M value and ADDR, while HbA1c level between groups was comparable (9.94 ± 2.79% vs. 9.70 ± 2.30%; *P* = 0.672). Above calculated results signified that HbA1c may be inferior to CGM metrics in building relationships with sudomotor dysfunction. Importantly, comparing groups with similar HbA1c values but different short-term glucose fluctuation values and ‘time in ranges’ may help determine the role of these dysglycemia components played in DPN. When it comes to people with T2D, former studies documented that TIR derived from data of CGM has a significantly positive correlation with peripheral nerve function [32], a significantly negative correlation with painful diabetic polyneuropathy [33]. Moreover, our previous work demonstrated a negative correlation between TIR and sudomotor dysfunction in T2D [19]. Consistent with those findings, we now observed that the prevalence of abnormal sudomoror function in the study population declined with the ascending of TIR tertiles. The linear and binary logistic regression analysis both indicated the independently negative association between TIR and sudomotor dysfunction even after adjusting for several risk factors.

Overnight glycemic control turns out to be a portion linked with the risk of diabetes outcomes. A prospective observational study was conducted on 162 pregnant women with gestational diabetes mellitus to investigate the association between temporal change of glucose and large for gestational age (LGA) infants [13]. By analysis of 7-day CGM data, the authors summarized that individuals who delivered LGA infants underwent significantly higher glucose concentrations during the 6-h period of the night (00:00–06:00 am) compared with mothers who did not have LGA infants (6.0 ± 1.0 mmol/L vs. 5.5 ± 0.8 mmol/L; *P* = 0.005) [13]. Another research found mean nocturnal glucose levels, rather than diurnal glucose values or glucose variability, were independently related to the seriousness of vascular remodeling [14]. For the first time, based on the results of data analysis, we draw a conclusion that overnight TIR assured of its place in sudomotor dysfunction. Possible explanations for patients with suboptimal overnight TIR can be considered; first, the overnight period lacks the glycemic excursions due to meals and exercise that occurs during the day. Second, under normal conditions of nighttime sleep, the elevated levels of growth hormone and cortisol cause the increment of the blood glucose [34]. Lastly, our included samples were patients who just came to the hospital for undesirable glycemic control in the past. One can saw this with the mean level of HbA1c was more than 9% in the study group.

Explaining exact mechanisms of the above scenario are difficult, however, we can try to understand it with rational speculation. Clinical evidences suggest that overnight glycaemic control in diabetes is susceptible to the daytime blood sugar levels and vise verse [35]. Poor nocturnal glycemic control can cause a reduced sensitivity of insulin at liver and other tissues, leading to fasting hyperglycemia [36], which is the cornerstone of all-round glycemic control throughout the day. So we guess that lower TIR in the overnight period exacerbates the whole-day TIR thereby exert an influence on diabetic outcomes. Anyway our result extends the clinical benefits of TIR by demonstrating that nocturnal TIR is also involved in diabetes outcomes.

Given that GV is involved in the natural process of DPN in subjects with T1D [37] and we used to verify in T2D that M value was contributed to sudomotor dysfunction [14], here we also analysed the effect that M value, ADDR, MAGE, SD and CV, an index of short-term GV, had on DPN, represented by abnormal sudomotor function. Again M value was proved to be a strong and stable predictive element for sudomotor dysfunction in T1D. By contrast, the other indicators are less effective predictors. ADDR was statistically associated with sudomotor dysfunction in the crude logistic model, but after adjustment of several confounding factors like sex, age, diabetes duration, BMI, Alb, smoking, BUN, DKD, and HbA1c, the relationship was inexistent with borderline significance. MAGE, SD and CV showed no relationship with sudomotor function firmly (data not shown).

Our study had a certain degree of limitation. In essence, this retrospective study cannot draw a cause-and-effect conclusion but can only describe the correlation between TIR and sudomotor function. Apart from that, all participants received 72 h of CGM, which perhaps made our results unrepresentative. Last but not least, despite relatively small number of samples in our cohort, patients with T1D are often regarded more homogeneous than patients with T2D. These people usually lack the other components of metabolic disorder so common for patients with T2D [38]. And we strictly conformed to inclusion and exclusion criteria to avoid the baseline characteristics of subjects vary enormously, which facilitates the chance of disclosing an association between CGM glucometrics and early diabetic complications in T1D.

## Conclusions

To sum up, TIR is negatively correlated with sudomotor dysfunction in T1D independent of HbA1c. Furthermore, decreased nocturnal TIR is more closely related to the impaired function of sudomotor nerves in sweat glands. Therefore, there is a reasonable prospect that optimizing TIR and, more importantly, highlighting the management of nocturnal TIR may help reduce rates of early DPN in T1D, yet prospective studies are awaited to further testify it.

## Data Availability

The datasets used and/or analysed during the current study are available from the corresponding author on reasonable request.

## Abbreviations

T1D: Type 1 diabetes
T2D: Type 2 diabetes
DPN: Diabetic peripheral neuropathy
CGM: Continuous glucose monitoring
DKD: Diabetic kidney disease
DR: Diabetic retinopathy
BMI: Body mass index
SBP: Systolic blood pressure
DBP: Diastolic blood pressure
BUN: Blood urea nitrogen
Scr: Serum creatinine
UA: Uric acid
TC: Total cholesterol
TG: Triglyceride
HDL: High-density lipoprotein
LDL: Low-density lipoprotein
Alb: Serum albumin
HbA1c: Hemoglobin A1c
MDSI: Multiple daily subcutaneous injection
TBR: Time below range
TIR: Time in range
TAR: Time above range
GV: Glucose variability
ADDR: Average daily risk range
MAGE: Amplitude of glucose excursions
SD: Standard deviation
CV: Coefficient of variation
AUC: Area under the curve
